# Multidimensional factors associated with childhood obesity severity in southern China: a cross-sectional study

**DOI:** 10.3389/fnut.2026.1942160

**Published:** 2026-09-29

**Authors:** Peifeng Zhuang, Bin Lin, Haiqing Li, Jinlu Zheng, Wenlin Tang, Wenbin Ye, Xiangjian Chen, Jingjing Wang

**Affiliations:** 1 Ningde Clinical Medical College of Fujian Medical University, Fuzhou, China; 2 Department of Joint Surgery and Sports Medicine, Ningde Municipal Hospital, Ningde, China; 3 Department of Pediatrics, Ningde Municipal Hospital, Ningde, China

**Keywords:** childhood obesity, multiple factors, obesity severity, prevention, Southeast China

## Abstract

**Background:**

Childhood obesity poses a major public health challenge, yet evidence on its graded associations with modifiable factors across dietary, behavioral, familial, and socioeconomic dimensions remains limited, particularly in Southeast China. This study aimed to comprehensively examine the multidimensional factors associated with childhood obesity.

**Methods:**

This cross-sectional study included 680 school-aged children from Fujian Province, China. Data on perinatal factors, family weight status, parental education, dietary intake, physical activity, and sleep behaviors were collected. Obesity severity was classified into four ordinal groups based on Chinese national standards. Ordinal logistic regression models were used to estimate associations. Group differences for categorical variables were assessed using the chi-square test.

**Results:**

Birth weight and infant feeding mode showed no significant association with obesity severity. Maternal care was associated with lower obesity risk in girls. Significant familial clustering was observed for paternal obesity (OR = 1.93, 95% CI: 1.20–3.06) and sibling obesity (OR = 2.26, 95% CI: 1.16–4.29). Lower parental education correlated with greater obesity severity. Breakfast frequency declined progressively with increasing obesity severity. At lunch and dinner, intakes of poultry, seafood, vegetables, fruits, and legumes decreased significantly across groups. Poultry intake at lunch was significantly associated with lower obesity risk (OR = 0.558, 95% CI: 0.354–0.878). Barbecue, cold drinks, and puffed foods were associated with increased obesity risk (OR = 1.763, 95% CI: 1.096–2.836). In contrast, nuts (OR = 0.477, 95% CI: 0.282–0.807) and biscuits (OR = 0.483, 95% CI: 0.322–0.723) showed significant were associated with lower risk. Regarding physical activity, ball sports (OR = 0.457, 95% CI: 0.227–0.918) and walking (OR = 0.368, 95% CI: 0.180–0.751) were significantly associated with lower obesity risk. Later bedtime and shorter sleep duration exhibited associations with obesity severity.

**Conclusions:**

Childhood obesity severity is associated with multiple factors, including familial weight status, parental education, meal diversity, snacking patterns, physical activity, and sleep behaviors. These findings may inform multifaceted, family centered approaches that could be considered for the prevention of childhood obesity.

## Graphical Abstract

**Figure:**
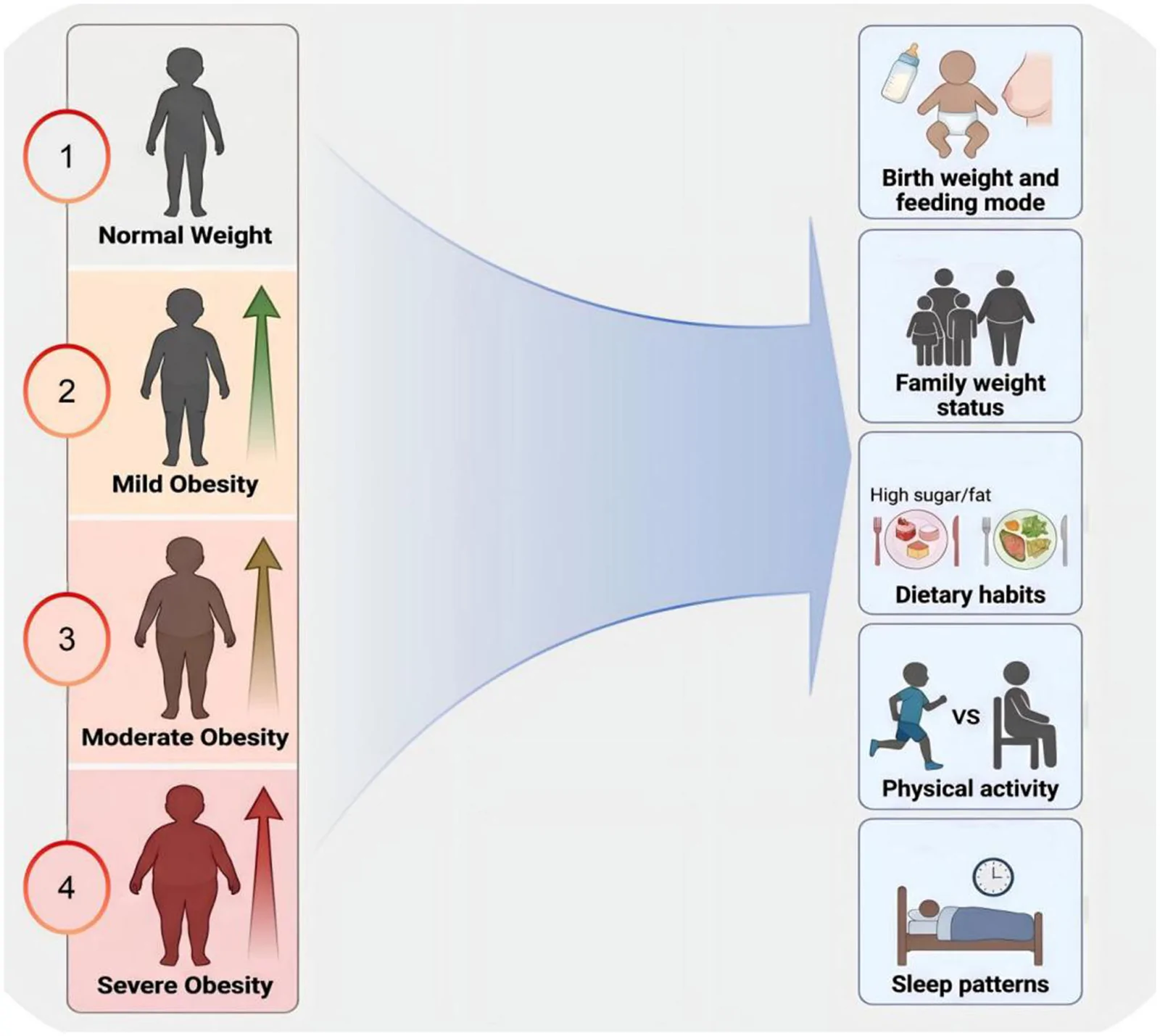

## Introduction

Childhood obesity has become one of the most pressing global public health challenges, affecting 38.9 million (5.7%) children under 5 years of age worldwide in 2020 and exerting long-term impacts on children's physical, psychological, and metabolic health (1). Early infant growth is closely associated with long term health outcomes, and rapid early weight gain increases the risk of coronary heart disease and diabetes (2–4).

A variety of factors affect children and adolescents. Evidence suggests that exclusive breastfeeding is inversely associated with lower childhood body mass index (BMI) and reduced risk of overweight compared with formula feeding (5–7). Children's eating behaviors and weight development are closely associated with family related factors. Beyond genetic predispositions, parental educational attainment, lifestyle habits, and parental guidance also exert significant influences (8, 9). As parents serve as behavioral role models, children often emulate their dietary patterns and physical activity habits, which significantly shape children's lifestyles and weight outcomes (10, 11). Household food availability and parental feeding practices also play a decisive role in children's eating behaviors. Healthy family meals and parental modeling of balanced diets are associated with improved dietary habits and reduced obesity risk in children (12). Furthermore, parenting styles characterized by emotional support, behavioral monitoring, and rule-based guidance further influence a range of obesity-related behaviors (13, 14).

Research has increasingly linked unhealthy lifestyle behaviors, such as insufficient sleep (15), poor sleep quality, reduced physical activity (16), and prolonged screen time (17–20) to a greater risk of weight gain and long term health consequences. Evidence from a longitudinal study indicates that unfavorable sleep habits at age one can significantly raise the likelihood of being overweight by age eight (21). Furthermore, suboptimal sleep may promote obesity by disrupting energy metabolism homeostasis (22). Nevertheless, the existing literature presents inconsistent findings. For example, although an inverse relationship between sleep duration and obesity has been reported (23), other investigations have not observed a significant link, potentially owing to small sample sizes or low rates of sleep problem reporting (24). Currently, how sleep-related variables, including duration, quality, and bedtime, interact remains largely unclear, especially among Chinese children, where systematic evidence is lacking (25).

We randomly surveyed over 600 children in Fujian of southern China to investigate the associations between infant feeding practices, birth weight, family members' weight status, dietary habits, exercise habits, as well as sleep habits and childhood obesity. Our findings revealed that childhood obesity is strongly associated with family members' weight status, suggesting a significant familial clustering of weight problems. In addition, dietary habits, snacking behaviors, physical activity, bedtime, and sleep duration were all found to have a measurable impact on childhood obesity. Specifically, unhealthy dietary patterns and insufficient physical activity were associated with a higher risk of excess weight. Moreover, both late bedtimes and shorter sleep duration emerged as associations, highlighting the importance of sleep related factors in weight management during childhood. These findings underscore the multifactorial nature of childhood obesity and suggest that prevention strategies should target not only individual behaviors but also the broader family environment.

## Method

### Study population

This school based cross sectional survey was conducted in the main urban area of Ningde City, Fujian Province, from March to July 2023. Using a stratified random sampling approach, we selected one primary school and one secondary school from each of the two districts (Jiaocheng and Dongqiao), all of which were public schools and agreed to participate. From each grade level (grades 1 to 9), one to two classes were randomly selected, and all children in the selected classes were invited to participate. A total of 1,509 children underwent physical examination, and complete epidemiological questionnaires were returned by the guardians of 680 children (344 boys and 336 girls, aged 6–16 years), yielding a response rate of 45.06%. Comparison between participants and non-participants revealed no significant differences in key demographic and anthropometric characteristics, suggesting that selection bias was minimal. The study sample is considered representative of urban Han Chinese children in southern China, although generalization to other regions or populations should be made with caution. The study protocol was approved by the Scientific Research Ethics Committee of Ningde Hospital (Approval No. 20220810) and the Ningde Education Bureau, and written informed consent was obtained from the guardians of all participating children.

### Grouping criteria

The diagnosis of obesity was based on the 2022 Expert Consensus on the Diagnosis, Assessment, and Management of Childhood Obesity in China developed by the Subspecialty Group of Endocrinology, Hereditary and Metabolic Diseases, the Society of Pediatrics, Chinese Medical Association, with sex and age specific BMI cut off values derived from Chinese national standards for children aged 6–18 years. To further classify obesity severity, we adopted the weight for height method recommended by the Expert Consensus on Medical Nutrition Therapy for Overweight/Obesity in China (2016 Edition), whereby the standard weight was defined as the 50th percentile weight of well-nourished children of the same height, with the normal range set at ±10% of the standard weight. Accordingly, overweight was defined as >15% above standard weight, mild obesity as >20%, moderate obesity as >30%, and severe obesity as >50% above the standard weight, allowing children to be classified into four ordinal groups: normal weight, mild obesity, moderate obesity, and severe obesity.

### Survey methods

Data were collected through two components: a school-based physical examination and a guardian reported epidemiological questionnaire. All anthropometric measurements, including height, weight, chest circumference, and hip circumference, were objectively measured by trained medical staff following standardized protocols, with systematic training provided prior to the survey on measurement techniques, BMI calculation, unified diagnostic criteria, questionnaire completion, and quality control procedures. The epidemiological questionnaire, completed by parents or primary caregivers, covered birth conditions, postnatal feeding practices, family health status, parental education level, and children's daily routines, dietary behaviors, physical activity, and sleep duration. Dietary information was collected using a validated food frequency questionnaire (FFQ) with a recall period of 6–12 months, which assessed consumption frequency of various food groups across breakfast, lunch, dinner, and snacks, although portion sizes and total energy intake were not quantified. Physical activity frequency and types, as well as bedtime and sleep duration, were assessed through structured caregiver reported items in the questionnaire.

### Statistical analysis

Descriptive statistics were presented as medians with interquartile ranges for continuous variables and as frequencies with percentages for categorical variables. Group comparisons across childhood obesity severity categories were performed using appropriate parametric or non-parametric tests. For categorical variables, the chi-square test was used to assess overall differences across multiple groups; when the overall test was significant, *post-hoc* pairwise comparisons between each obesity group and the normal weight group were conducted with Bonferroni correction. Correlation analysis, including correlation matrices, was employed to examine relationships between snack intake patterns and to identify clustering of healthy and unhealthy snacks.

To evaluate the independent associations between various exposure factors and childhood obesity severity, we fitted a series of ordinal logistic regression models (proportional odds models), as the outcome variable comprised four ordered categories (normal, mild, moderate, and severe obesity). Separate models were constructed for each exposure domain, with covariates adjusted according to the specific research question and detailed in the respective figure legends. In all models, the staple food category was excluded due to near zero variation (>98% consumption). Multicollinearity was assessed using the variance inflation factor (VIF), and the proportional odds assumption was verified using the Brant test for each model. Results were presented as odds ratios (ORs) with 95% confidence intervals (CIs), and forest plots were generated to visualize these associations. All statistical tests were two sided, and a *P-*value < 0.05 was considered statistically significant. Statistical analyses were performed using R software (version 4.2.2).

## Results

### Perinatal and early postnatal factors affecting childhood obesity severity

Birth weight distributions overlapped substantially across the four obesity severity groups, with comparable median values and ranges of variation (Figure 1A). Correlation analysis revealed a weak positive association between birth weight and the probability of obesity (Figure 1B), which did not reach statistical significance. These findings suggest that, in the present study population, birth weight is not significantly associated of childhood obesity severity.

**Figure 1:**
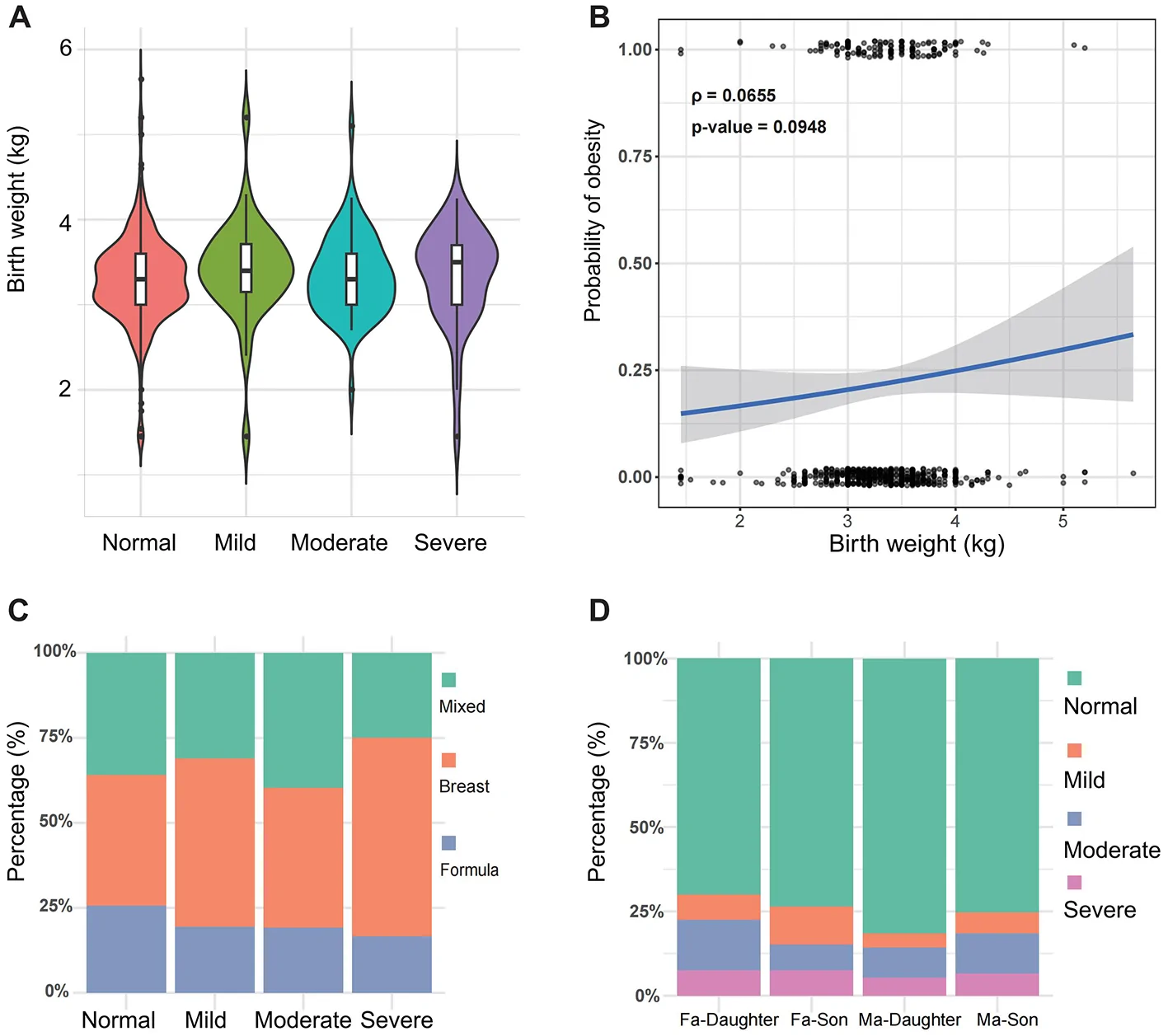
Perinatal and early postnatal factors associated with childhood obesity severity. **(A)** Box plots showing birth weight distributions across the normal, mild, moderate, and severe obesity groups. Median values and interquartile ranges were similar across all four groups. **(B)** Scatter plot illustrating the correlation between birth weight and the probability of obesity. **(C)** Bar charts displaying the proportions of infant feeding practices (mixed feeding, breastfeeding, and formula feeding) across obesity severity groups. **(D)** Prevalence of normal weight and obesity (combined mild, moderate, and severe) among different caregiver–child dyads.

Across all obesity severity groups, mixed feeding and breastfeeding were the predominant infant feeding practices, while formula feeding accounted for the smallest proportion (approximately 20%). Notably, the proportion of breastfeeding showed an increasing trend with greater obesity severity, rising from approximately 40% in the Normal group to about 55% in the Severe group, accompanied by a corresponding decrease in formula feeding. Mixed feeding accounted for the smallest proportion among children with severe obesity, while it remained relatively stable across the other three groups (Figure 1C). Although statistical analyses revealed no significant differences between groups, this trend was evident.

Normal-weight children constituted the majority (70%−80%) across all caregiver–child dyads. A key finding was that the combined prevalence of obesity was significantly lower among daughters cared for by their mothers compared to other caregiver–child combinations, suggesting a potential protective association against childhood obesity in girls. This protective effect was not observed for sons cared for by their mothers, nor for children of either sex cared for by their fathers (Figure 1D).

This study indicates that birth weight and infant feeding mode are not significantly correlated of childhood obesity severity. Maternal care was significantly associated with lower obesity risk for girls, suggesting that the interaction between sex and caregiver should be taken into account in future intervention strategies.

### Family related factors associated with childhood obesity

With respect to the association between family members' weight status and childhood obesity risk, the distribution characteristics showed that among all relative categories, the proportion of severe obesity was the highest in the father group, which was significantly higher than that in the normal weight group (Figure 2A). In addition, the proportions of moderate to severe obesity in the mother and sibling groups were also significantly higher than those in the normal weight group (Figure 2A), indicating a family aggregation tendency of obesity.

**Figure 2:**
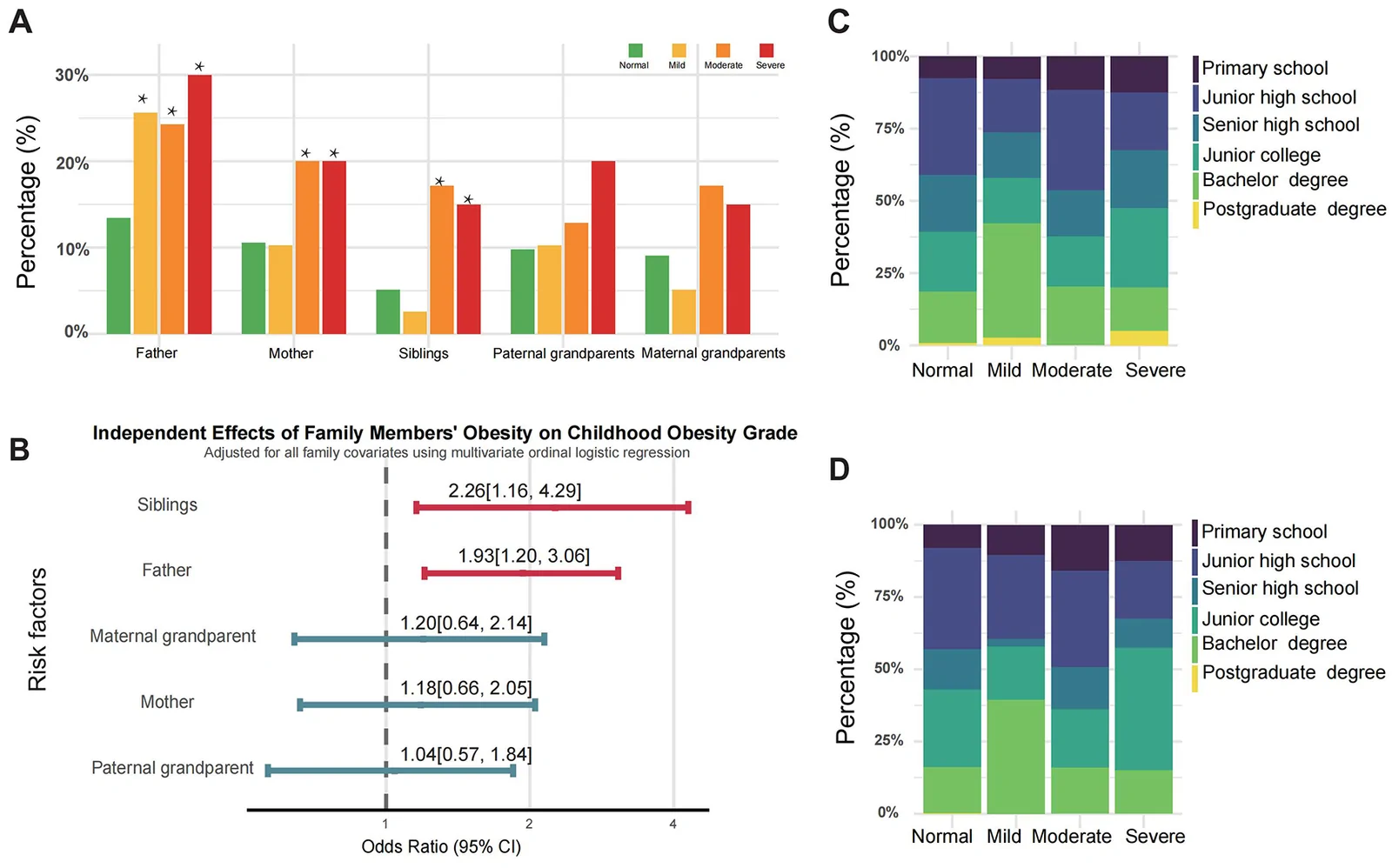
Family-related factors associated with childhood obesity severity. **(A)** Proportion of obesity among family members (father, mother, siblings, paternal grandparents, and maternal grandparents) across childhood obesity severity groups (normal, mild, moderate, severe). **(B)** Forest plot showing odds ratios (ORs) and 95% confidence intervals (CIs) for the association between obesity in family members and childhood obesity risk. **(C)** Father's educational background across childhood obesity severity groups. **(D)** Mother's educational background across childhood obesity severity group. * Indicates *p* < 0.05.

The forest plot analysis revealed that obesity of siblings (OR = 2.26, 95% CI: 1.16–4.29), and fathers (OR = 1.93, 95% CI: 1.20–3.06) was significantly positively associated with childhood obesity risk (Figure 2B). These findings suggest that obesity of core family members shows a significant positive association for childhood obesity. However, there was no significant statistical association between the obesity of mother, paternal or maternal grandparents and childhood obesity (Figure 2B).

Regarding parental educational background and childhood obesity severity, maternal educational background showed a gradient trend similar to that of paternal educational background (Figures 2C, D). As childhood obesity severity increased from normal to moderate, the proportion of fathers with primary school education increased, while the proportion of those with Junior high school or above decreased correspondingly (Figure 2C). Overall, these results suggest an inverse association between parental educational attainment and childhood obesity severity.

In summary, there was a family aggregation effect of obesity. The obese status of core family members (fathers and siblings) was significantly positively associated with childhood obesity risk, among which siblings had the strongest impact. This indicates that genetics and family lifestyle are jointly associated with the occurrence of childhood obesity. A socioeconomic gradient was also observed. The lower the parental educational level, the more severe the childhood obesity, reflecting that educational background may indirectly affect children's weight status by influencing feeding habits, lifestyles, and other pathways. Additionally, grandparents had a limited role, which obesity was not significantly associated with childhood obesity risk, suggesting that the nuclear family may be a key environmental unit associated with childhood obesity.

### Breakfast frequency and food types among children with different obesity severity

Regarding breakfast frequency, as childhood obesity severity increased from normal to mild, moderate, and severe, the proportion of children who consumed breakfast daily showed a gradual decreasing trend (Figure 3A), whereas the proportion of those who never or only occasionally ate breakfast increased progressively (Figure 3B). Compared with the normal weight group, children with moderate and severe obesity had significantly lower daily breakfast frequency and more irregular breakfast habits.

**Figure 3:**
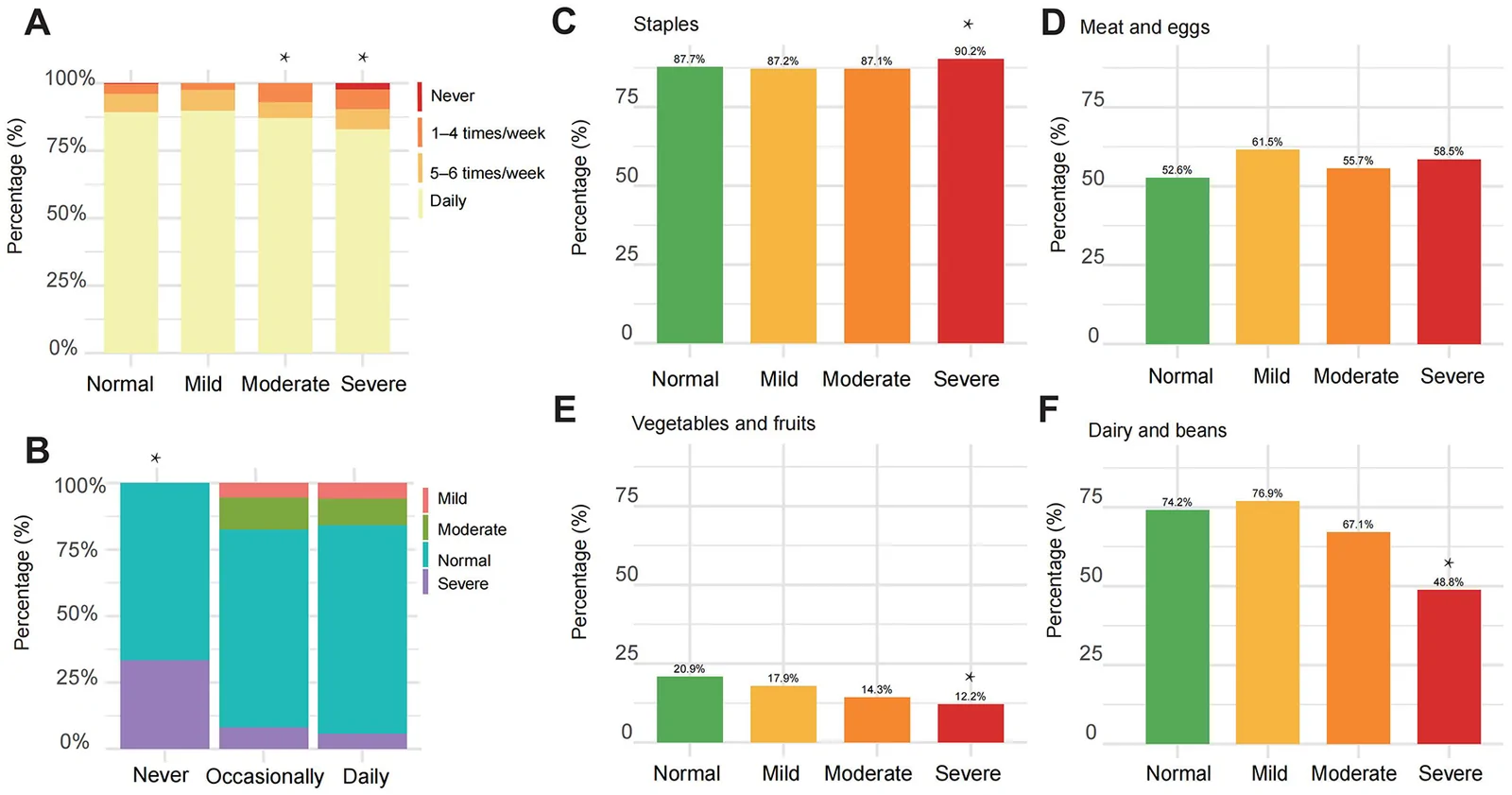
Breakfast frequency and food types across childhood obesity severity groups. **(A)** Proportion of children consuming breakfast daily from normal to mild, moderate, and severe. **(B)** Proportion of children who never or only occasionally ate breakfast. **(C)** Staple food intake at breakfast. **(D)** Meat and egg intake at breakfast. **(E)** Vegetable and fruit intake at breakfast. **(F)** Dairy and bean intake at breakfast. * Indicates *p* < 0.05.

In terms of breakfast food types, staple food intake accounted for a relatively high proportion across all groups. The proportion of staple food intake was significantly higher in the severe obesity group than in the other groups (Figure 3C), indicating that staple food consumption increased with worsening childhood obesity. The proportion of meat and egg intake exhibited an upward trend, with all obese groups showing higher proportions than the normal weight group (Figure 3D). In contrast, the proportion of vegetable and fruit intake decreased significantly as obesity severity increased; the normal-weight group had the highest proportion, and the severe obesity group the lowest, with the differences reaching statistical significance (Figure 3E). Similarly, the proportion of dairy and bean intake decreased, and the severe obesity group had a significantly lower proportion than other groups (Figure 3F).

These findings indicate that childhood obesity severity is closely associated with breakfast behaviors, with greater obesity severity correlates with lower breakfast frequency and a breakfast composition higher in staple foods and meat/eggs, but lower in vegetables, fruits, dairy products, and beans. Regular breakfast consumption and a breakfast pattern rich in vegetables, fruits, dairy products, and beans may be associated with lower odds against childhood obesity.

### Lunch variety and food types among children with different obesity severity

The distribution of lunch food group intake across different obesity severity groups revealed several notable patterns. Staple food consumption was nearly universal across all groups, with proportions ranging from 92.0% to 100.0%, and the mild obesity group exhibited the highest proportion at 100.0% (Figure 4A), indicating no clear gradient association between staple intake and obesity severity. In contrast, the intake of poultry, seafood, vegetables and fruits, and legumes showed a consistent decreasing trend as obesity severity increased (Figures 4B–F). The normal weight group had the highest proportions of poultry at 81.5%, seafood at 64.0%, vegetables and fruits at 76.4%, and legumes at 47.2%. These proportions declined progressively in the moderate and severe obesity groups, with the severe group reaching 61.0% for poultry, 56.1% for seafood, 65.9% for vegetables and fruits, and 31.7% for legumes. Between group differences for poultry, vegetables and fruits, and legumes were statistically significant. Egg intake showed a different pattern, as the mild obesity group had the highest proportion at 76.9%, followed by the normal weight group at 64.7%, while the severe obesity group had the lowest at 46.3% (Figures 4B–F).

**Figure 4:**
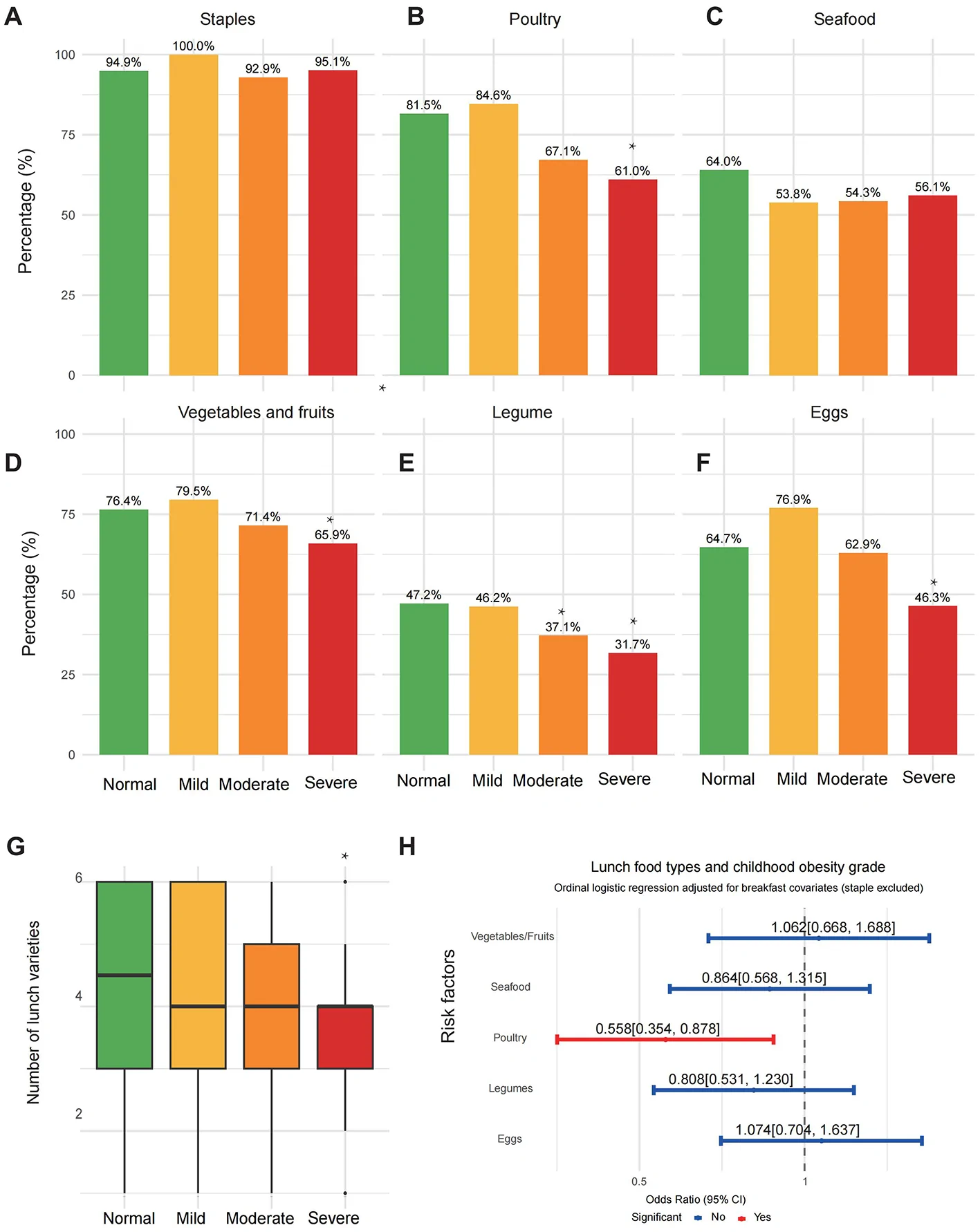
Lunch food group intake, dietary diversity, and associations with childhood obesity severity. **(A–F)** Intake of staple food, poultry, seafood, vegetables and fruits, legumes, and eggs across obesity severity groups. **(G)** Box plot showing the number of food types consumed at lunch. **(H)** Forest plot of odds ratios (ORs) and 95% confidence intervals (CIs) for the association between specific lunch food groups and childhood obesity risk. * Indicates *p* < 0.05.

Regarding the number of food types consumed at lunch, box plot analysis indicated a declining trend in lunchtime dietary diversity with increasing obesity severity. The normal weight and mild obesity groups had the highest median number of food types at approximately four, followed by the moderate obesity group, while the severe obesity group had the lowest median at approximately three to four (Figure 4G). This finding suggests that more severe childhood obesity is associated with a more monotonous lunch dietary pattern characterized by fewer food categories.

The association between specific lunch food groups and childhood obesity risk was further examined using forest plot analysis. Poultry intake was significantly and negatively associated with obesity risk, with an odds ratio of 0.558 and a 95% confidence interval of 0.354–0.878, identifying poultry as being negatively associated with obesity risk against childhood obesity. Legumes and seafood also exhibited odds ratios below 1, indicating a protective trend in which higher intake of these foods tended to be associated with lower obesity risk, although these associations did not reach statistical significance. In contrast, and vegetables/fruits and eggs showed odds ratios slightly above 1, suggesting a weak positive association with obesity risk, but neither was statistically significant (Figure 4H).

In conclusion, the severity of childhood obesity is associated with a distinct lunch intake gradient. As obesity severity increases, the consumption of poultry, seafood, vegetables, fruits, and legumes, which are foods rich in high quality protein and dietary fiber, declines significantly, whereas staple food intake remains high across all groups. Moreover, greater obesity severity correlates with poorer lunchtime dietary diversity, as reflected by a reduced number of food types. Poultry intake showed a significant negative association, and legumes and seafood showed consistent protective trends. These findings support the notion that increasing the intake of these food groups and enhancing lunchtime dietary variety may be relevant to prevention efforts of childhood obesity.

### Dinner variety and food types among children with different obesity severity

The distribution of dinner food group intake across obesity severity groups showed that staple food consumption was extremely high in all groups, ranging from 95.7% to 97.6%, with the severe obesity group having the highest proportion (Figure 5A). This indicates that staple food intake is universal and shows no clear negative association with obesity severity. In contrast, the intake of poultry, seafood, legumes, and eggs declined significantly as obesity severity increased. The normal weight group had the highest proportions of poultry at 74.2%, seafood at 61.9%, and legumes at 45.5%, while the severe obesity group had the lowest proportions at 53.7% for poultry, 43.9% for seafood, and 31.7% for legumes. Between group differences for poultry, seafood, and legumes were statistically significant (Figures 5B–E). Egg intake followed a different pattern, with the mild obesity group having the highest proportion at 79.5%, followed by the normal weight group at 60.4%, and the severe obesity group the lowest at 39.0%, with significant differences with other groups (Figure 5F). Regarding vegetable and fruit intake at dinner, the overall proportion remained high across all obesity severity groups, ranging from 74.3% to 79.5%, with only minor differences between groups. The mild obesity group showed the highest intake proportion at 79.5%, followed by the normal weight group at 78.7%, whereas the moderate obesity group had the lowest proportion at 74.3% and the severe obesity group at 75.6%. No statistically significant differences were detected among the groups, indicating that vegetable and fruit intake at dinner does not follow a clear gradient trend with increasing severity of childhood obesity (Figure 5D).

**Figure 5:**
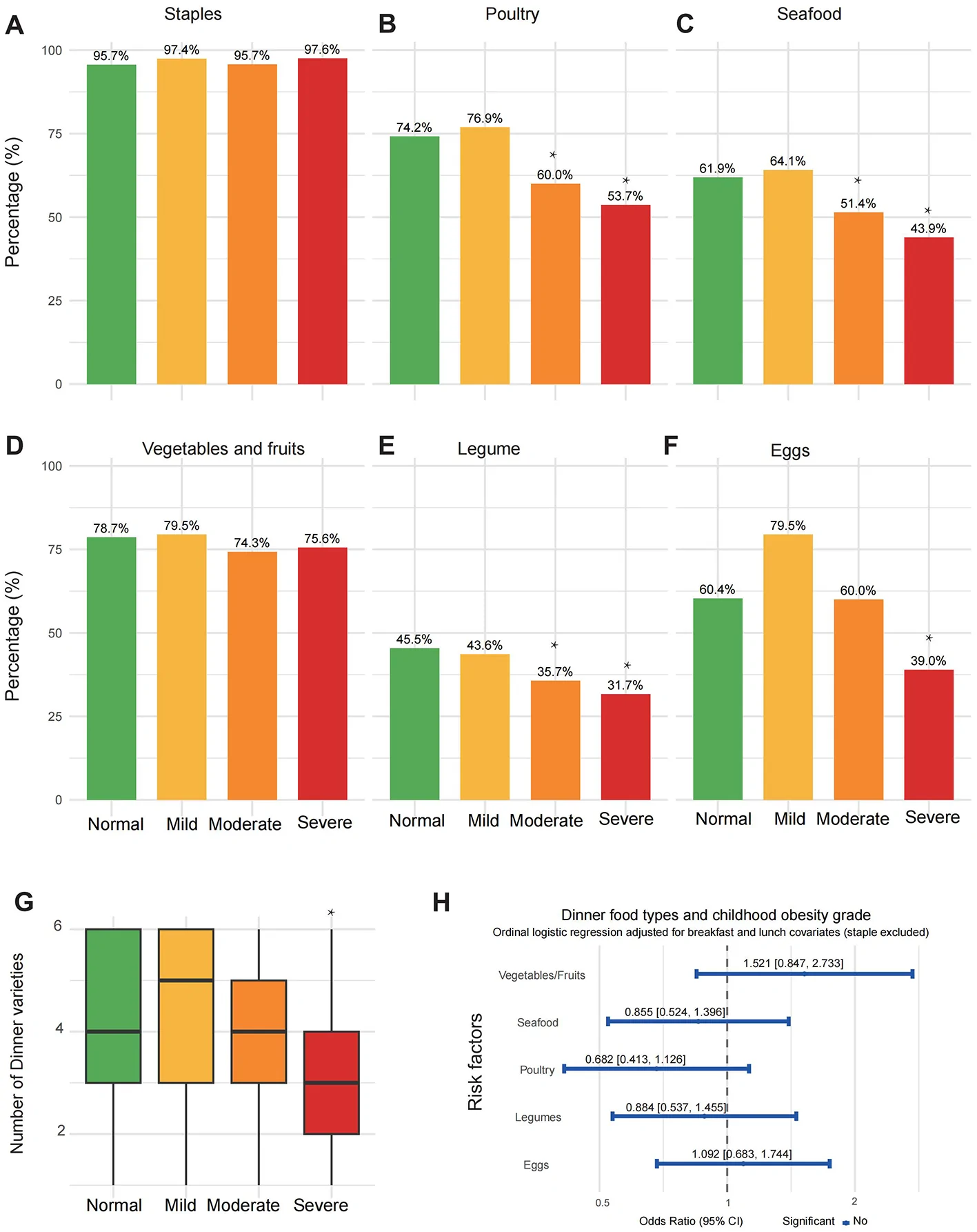
Dinner food group intake, dietary diversity, and associations with childhood obesity severity. **(A–F)** Intake of Staple food, poultry, seafood, legumes, eggs, and vegetables/fruits at dinner across obesity severity groups. **(G)** Box plot showing the number of food types consumed at dinner. **(H)** Forest plot of odds ratios (ORs) and 95% confidence intervals (CIs) for the association between specific dinner food groups and childhood obesity risk. * Indicates *p* < 0.05.

Regarding the number of food types consumed at dinner, box plot analysis revealed a clear decreasing trend in dietary diversity as obesity severity increased. The normal weight and mild obesity groups had the highest median number of food types, approximately four to five, followed by the moderate obesity group, while the severe obesity group had the lowest median at approximately three, with significant differences between groups (Figure 5G). This finding indicates that greater obesity severity is associated with poorer dinner dietary diversity, characterized by a more monotonous meal pattern.

Forest plot analysis revealed that none of the dinner food groups reached statistical significance (all 95% CIs crossed 1). Nevertheless, poultry (OR = 0.682), seafood (OR = 0.855), and legumes (OR = 0.884) exhibited protective tendencies, whereas vegetables/fruits (OR = 1.521) and eggs (OR = 1.092) showed weak risk tendencies (Figure 5H). These non-significant but directional findings suggest that certain dinner food choices may warrant further exploration in larger cohorts.

In conclusion, as childhood obesity severity increases, the intake proportions of poultry, seafood, legumes, and eggs, which are rich in high quality protein and dietary fiber, decline significantly, whereas staple food intake remains high across all groups. Greater obesity severity correlates with poorer dinner dietary diversity, with the severe obesity group showing a significantly reduced number of food types. Poultry, seafood, and legumes each demonstrated protective directions against childhood obesity, although none of these associations reached statistical significance. These findings suggest that increasing the consumption of these food groups and enhancing dinner dietary variety may help prevent childhood obesity.

### Snack consumption patterns, gradients, and associations with childhood obesity severity

Correlation analysis of snack intake revealed two distinct clusters. A cluster of unhealthy snacks, including preserved products, sugar sweetened beverages, and barbecue, cold drinks, and puffed foods, showed moderate positive correlations with each other, with correlation coefficients ranging from 0.24 to 0.36 (Figure 6A). This indicates that these high sugar, high fat, and high salt snacks tend to be consumed together. A second cluster of healthier snacks, comprising dairy products, nuts, and fruits, exhibited weak positive correlations, with coefficients between 0.11 and 0.22, suggesting a relatively healthy snack combination (Figure 6A). Moreover, correlations between healthy and unhealthy snack groups were mostly weakly negative or near zero, implying a certain degree of mutual exclusivity in snacking behavior.

**Figure 6:**
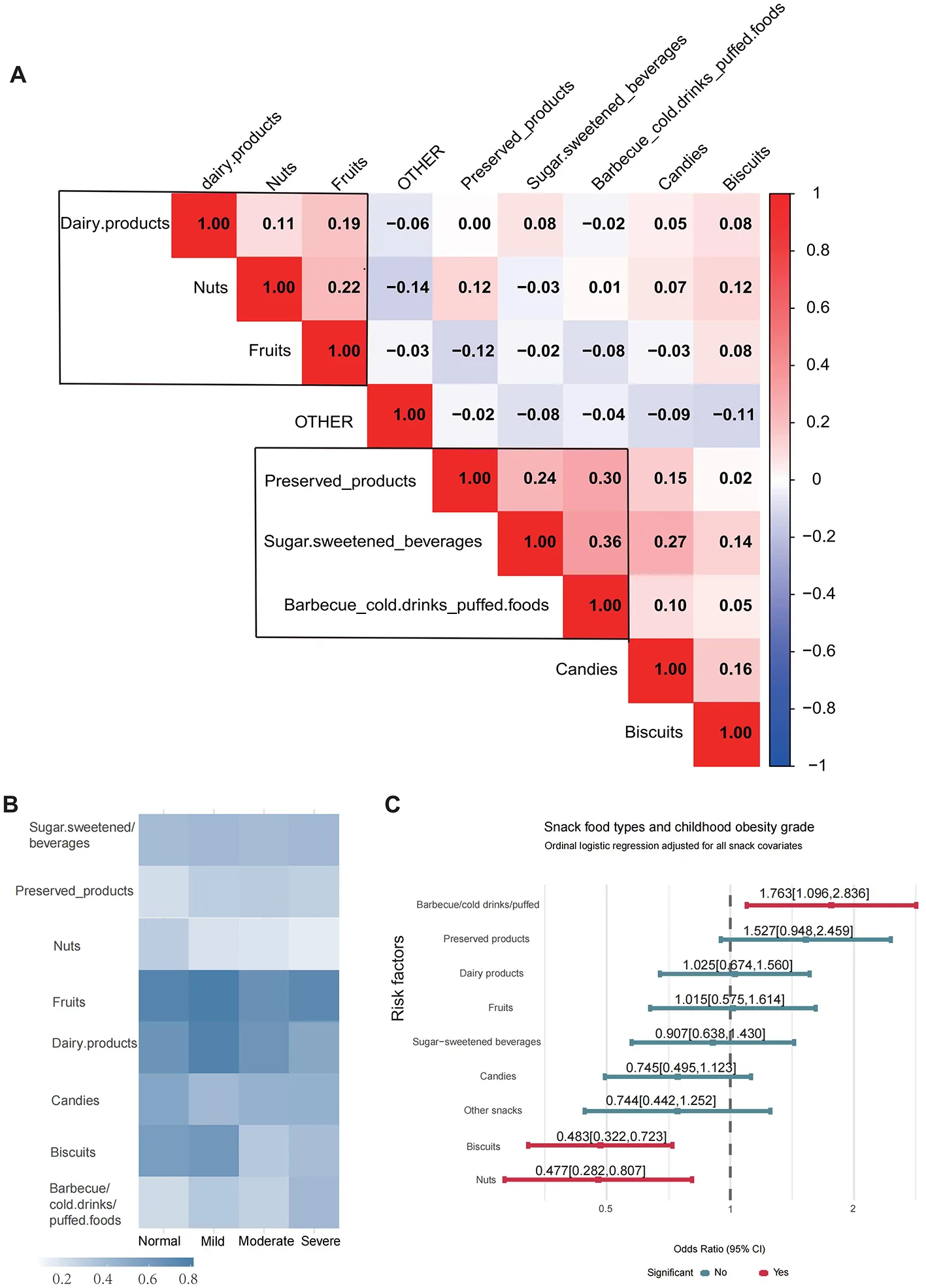
Snack consumption patterns, gradients, and associations with childhood obesity severity. **(A)** Correlation matrix of snack intake. **(B)** Gradient distribution of snack intake across obesity severity groups. **(C)** Forest plot of odds ratios (ORs) and 95% confidence intervals (CIs) for the association between snack types and childhood obesity risk.

Regarding the gradient distribution of snack intake across obesity severity groups, fruit intake remained high in all groups with little variation, showing no clear decline as obesity severity increased (Figure 6B). Dairy product intake was overall the highest among snack categories, with only a slight decrease in the severe obesity group (Figure 6B). Nut intake was highest in the normal weight group and gradually decreased with increasing obesity severity, reaching the lowest level in the severe group (Figure 6B). Among unhealthy snacks, sugar sweetened beverage intake was consistently high across groups without significant between group differences. Preserved product intake was lowest in the normal weight group and highest in the obesity groups (Figure 6B). Barbecue, cold drinks, and puffed foods showed the lowest intake in the normal weight group, relatively higher intake in the obesity groups, and the highest intake in the severe obesity group. Candy and biscuit intake showed minimal variation across groups, with candy intake slightly higher in the normal weight group and biscuit intake relatively high in the normal and mild obesity groups (Figure 6B).

The association between snack types and childhood obesity risk was examined using forest plot analysis (Figure 6C). Among the snack categories, barbecue, cold drinks, and puffed foods were significantly positively associated with obesity risk (OR = 1.763, 95% CI: 1.096–2.836), whereas nuts (OR = 0.477, 95% CI: 0.282–0.807) and biscuits (OR = 0.483, 95% CI: 0.322–0.723) demonstrated significant were associated with lower obesity risk. Preserved products showed a risk tendency (OR = 1.527, 95% CI 0.948 to 2.459). Dairy products (OR = 1.025, 95% CI 0.674 to 1.560) and fruits (OR = 1.015, 95% CI 0.638 to 1.614) essentially showed no appreciable association. Candies (OR = 0.907, 95% CI 0.575 to 1.430), sugar-sweetened beverages (OR = 0.745, 95% CI 0.495 to 1.123), and other snacks (OR = 0.744, 95% CI 0.442 to 1.252) exhibited protective tendencies, although none reached statistical significance (Figure 6C). These findings suggest that the association between snack consumption and childhood obesity is heterogeneous, with both risk-enhancing and protective patterns observed across different snack types.

The present analysis identified two distinct snack consumption patterns among children: a “healthy pattern” characterized by the intake of nuts, fruits, and dairy products, and an “unhealthy pattern” characterized by the intake of barbecue/cold drinks/puffed foods, preserved products, and sugar-sweetened beverages. The correlation matrix revealed moderate positive correlations within the unhealthy pattern (Figure 6A). Notably, barbecue/cold drinks/puffed foods were positively correlated with preserved products (*r* = 0.30) and sugar-sweetened beverages (*r* = 0.36), suggesting a clustering of unhealthy snack choices. In contrast, nuts and fruits showed a moderate positive correlation (*r* = 0.22), indicating a tendency toward healthier snack combinations, with some degree of mutual exclusivity between the two patterns.

### Physical activity types, frequency, and associations with childhood obesity severity

To examine the association between physical activity frequency and obesity severity, we analyzed its distribution across different weight groups, as shown in Figure 7A. Across all groups, the majority of children exercised 1–3 times per week, with proportions ranging from 61.4% (moderate obesity) to 71.8% (mild obesity). The proportion of inactive children was highest in the moderate obesity group (28.6%) and lowest in the normal-weight group (11.7%). Daily exercise participation was consistently low across all groups (4.3%−10.0%), as was exercise 4–6 times per week (4.9%−10.9%). The mild obesity group showed a distribution pattern similar to that of normal weight children, with a high proportion of regular exercise (71.8%) and a low inactivity rate (12.8%). Chi-square test confirmed a significant overall difference across groups (χ^2^ = 24.87, df = 9, *P* = 0.003). *Post-hoc* comparisons indicated that the mild obesity group did not differ significantly from the normal weight group (*P* = 0.843), whereas the moderate and severe obesity groups had significantly higher inactivity rates and lower regular exercise participation. Notably, the moderate obesity group had the highest inactivity rate (28.6%), suggesting that moderate obesity may represent a critical turning point for declining physical activity. Overall, a clear inverse gradient was observed, with physical activity frequency decreasing as obesity severity increased, particularly from the moderate stage onward.

**Figure 7:**
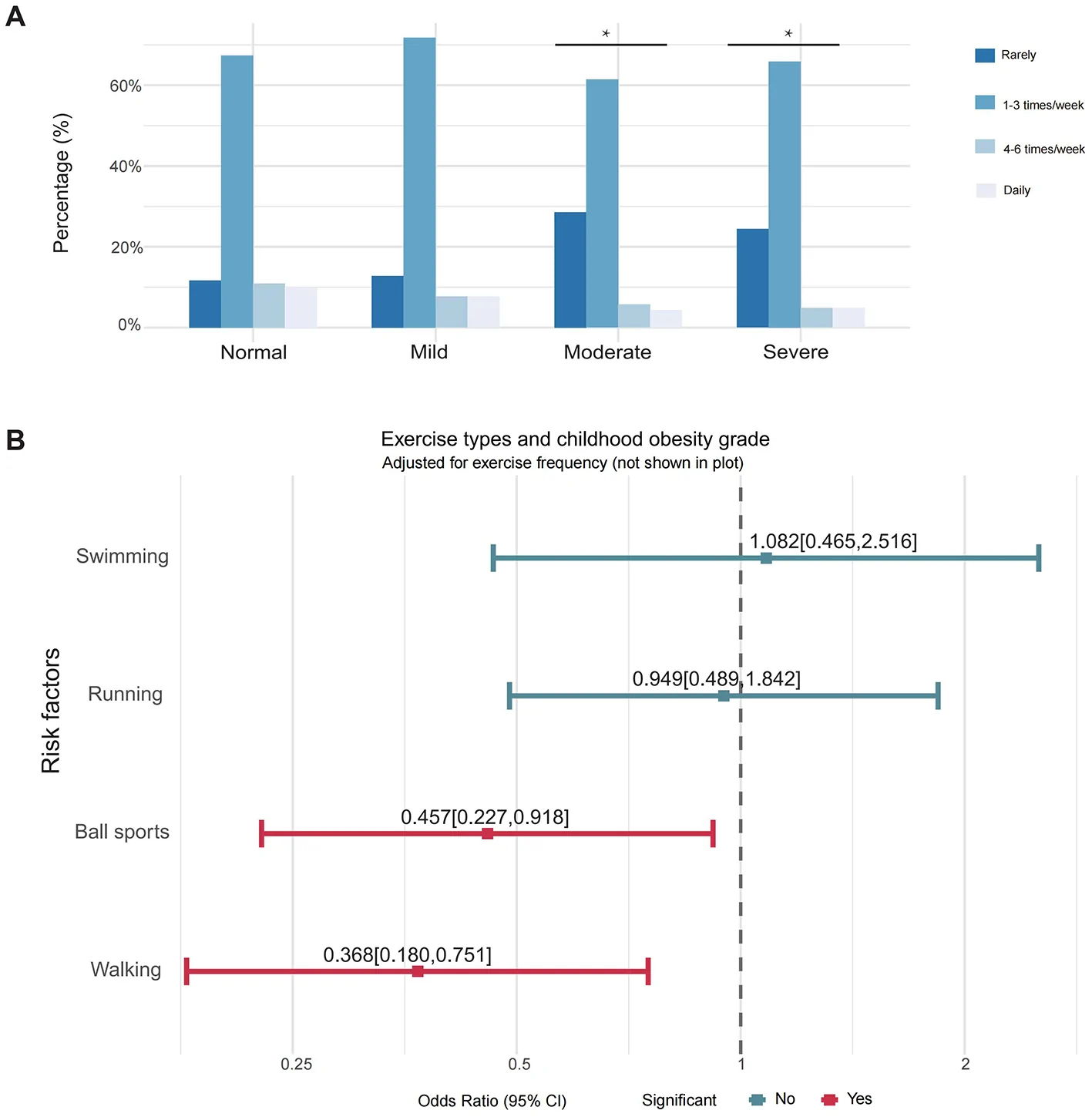
Physical activity frequency, and types associations with childhood obesity severity. **(A)** Physical activity frequency across obesity severity groups. **(B)** Forest plot of odds ratios (ORs) and 95% confidence intervals (CIs) for the association between physical activity types and childhood obesity risk. * Indicates *p* < 0.05.

The association between physical activity types and childhood obesity risk was examined using forest plot analysis (Figure 7B). After adjustment for exercise frequency, ball sports (OR = 0.457, 95% CI: 0.227–0.918) and walking (OR = 0.368, 95% CI: 0.180–0.751) were significantly and negatively associated with obesity risk, identifying these two activity types as significant protective factors against childhood obesity. Running also demonstrated a protective tendency (OR = 0.949, 95% CI: 0.489–1.842), although this association did not reach statistical significance. Swimming showed no clear protective effect (OR = 1.082, 95% CI: 0.465–2.516), with its confidence interval crossing the null (Figure 7B).

In summary, both the frequency and type of physical activity are differentially associated with obesity severity. The decline in activity appears to become most pronounced from moderate obesity onward, and interventions promoting ball sports and walking may be particularly beneficial for obesity prevention.

### Bedtime, sleep duration, and associations with childhood obesity severity

The distribution of bedtime across obesity severity groups revealed a clear gradient pattern. In the normal weight group, 24% of children went to bed between 10 and 12 o'clock, while the majority went to bed between 9 and 10 o'clock, and almost none stayed up past 12 o'clock. In the mild and moderate obesity groups, the proportion of children going to bed between 10 and 12 o'clock increased to approximately 35%, with very few staying up past midnight. In contrast, the severe obesity group had the highest proportion of children going to bed between 10 and 12 o'clock at approximately 43%, and a small proportion of children stayed up past 12 o'clock (Figure 8A). Between group differences were statistically significant. Overall, greater obesity severity was associated with later bedtimes and a greater tendency to stay up late.

**Figure 8:**
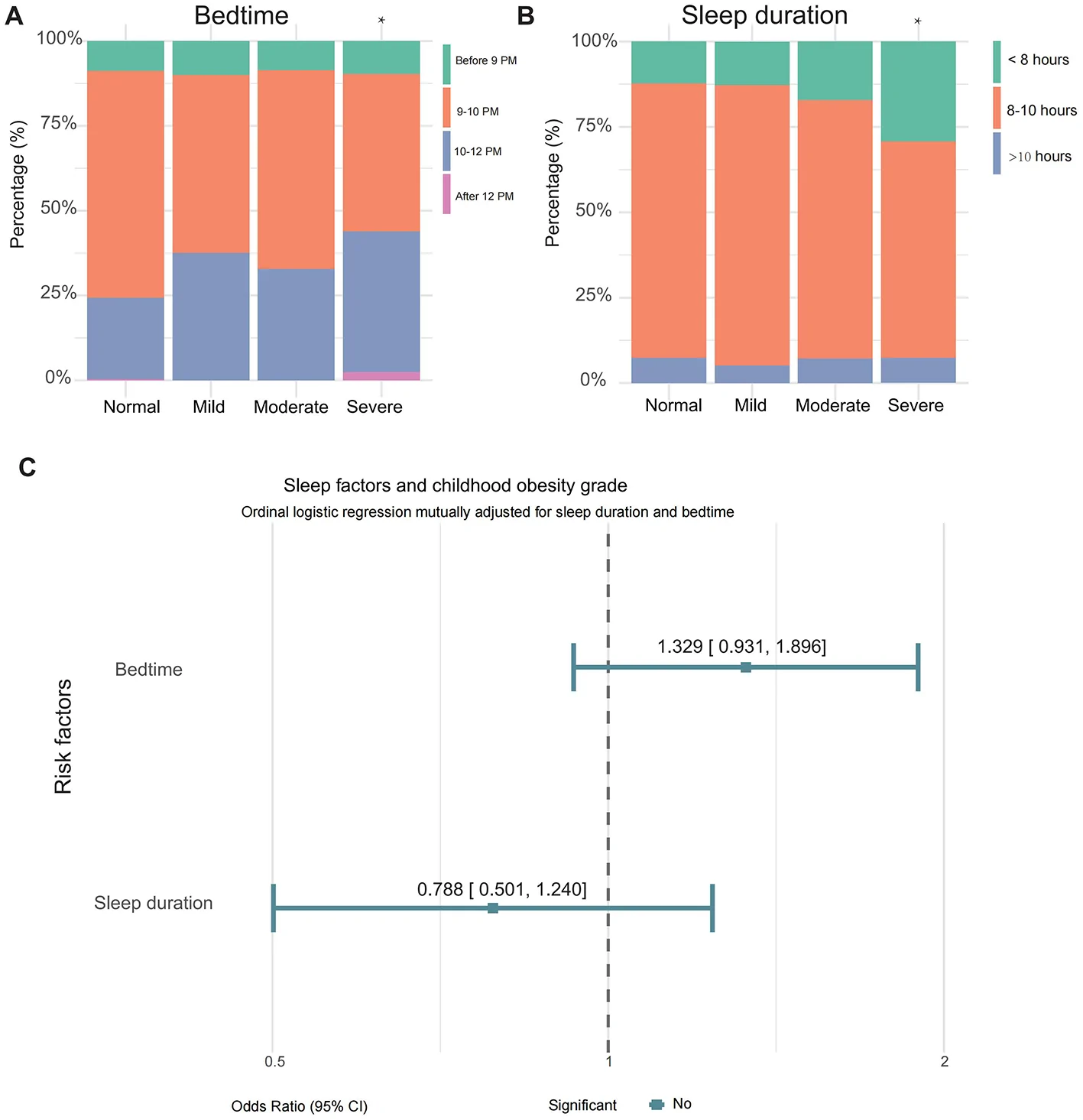
Bedtime, sleep duration, and associations with childhood obesity severity. **(A)** Distribution of bedtime across obesity severity groups. **(B)** Distribution of sleep duration across obesity severity groups. **(C)** Forest plot of odds ratios (ORs) and 95% confidence intervals (CIs) for the association between sleep indicators and childhood obesity risk. * Indicates *p* < 0.05.

Regarding sleep duration, the normal weight group had approximately 7% of children sleeping more than 10 h, with the majority sleeping 8–10 h. In the mild and moderate obesity groups, the proportion of children sleeping more than 10 h decreased slightly, while the proportion sleeping less than 8 h increased slightly. The severe obesity group had the highest proportion of children sleeping less than 8 h, with between group differences reaching statistical significance (Figure 8B). These findings demonstrate a gradient pattern in which greater obesity severity is associated with shorter sleep duration and a higher prevalence of insufficient sleep.

The association between sleep indicators and childhood obesity risk was examined using forest plot analysis (Figure 8C). Although neither bedtime nor sleep duration reached statistical significance, both variables exhibited directional trends consistent with expectations. Sleep duration demonstrated a protective tendency, with the point estimate suggesting that longer sleep duration may be associated with a lower risk of obesity, whereas later bedtime showed a trend in the opposite direction. Although these observed trends were not statistically robust, they may warrant further investigation in future studies with larger sample sizes and more objective sleep measurements.

In conclusion, a clear sleep pattern gradient was observed. As childhood obesity severity increased, bedtime was significantly delayed and sleep duration was significantly shortened, with marked increases in the proportions of late bedtime and insufficient sleep. Late bedtime and staying up late showed independent associations for childhood obesity, while adequate sleep duration showed a potential protective association. Regular sleep schedules, avoiding late nights, and ensuring sufficient sleep may represent important lifestyle targets for the prevention and intervention of childhood obesity.

## Discussion

This cross-sectional survey of over 600 children in Fujian Province systematically revealed graded associations between childhood obesity severity and multidimensional factors. Key findings included a significant familial clustering of obesity. The weight status of core family members, including father and siblings, showed a strong positive association for childhood obesity, with siblings exerting the strongest influence. Lower parental education level was associated with greater childhood obesity severity. At the behavioral level, lower breakfast frequency, reduced intake of poultry, seafood, vegetables, fruits, and legumes across three main meals, a snacking pattern shifting toward high sugar, high fat, and high salt options, lack of running and other physical activities, as well as delayed bedtime and shorter sleep duration, all exhibited clear graded relationships with obesity severity. These results are consistent with a multifactorial model of childhood obesity and suggest that stratified interventions targeting both the nuclear family environment and specific behavioral determinants may warrant further investigation.

Importantly, the cross-sectional design of this study precludes any inference of causality. All associations reported herein represent statistical correlations observed at a single time point, and the directionality of these relationships cannot be established. For example, while running showed a negative association with obesity severity, it remains equally plausible that children with pre-existing obesity may be less likely to engage in running due to physical discomfort, reduced exercise self-efficacy, or weight related stigmatization, rather than running causally reducing obesity risk. Similarly, the observed association between delayed bedtime and greater obesity severity could be partially explained by reverse causality, whereby obesity related physiological or psychological factors may disrupt sleep patterns. Therefore, the terms “risk” and “protective” are used throughout this manuscript strictly in an epidemiological sense to denote statistical associations, not causal effects. Future longitudinal cohort studies or randomized controlled trials are warranted to confirm the temporal sequence and potential causal pathways underlying these observed associations.

The present study found that obesity status of fathers and siblings was all significantly positively associated with childhood obesity, with siblings showing the highest odds ratio (Figures 2A, B). This finding is highly consistent with the previous results (26). Compared with children who did not have an obese older child at home, those who co-resided with an obese older child had a significantly higher risk of obesity. This association remained robust after adjusting for confounders such as socioeconomic status, age, and sex, suggesting that the obesity status of older siblings within the family may have an independent association on the obesity risk of younger children through pathways involving lifestyle and behavioral habits. A study within the European IDEFICS project found that, compared with only children, having one sibling was associated with approximately 26% lower risk of overweight or obesity, and the risk was even lower for children with two or more siblings (33). These previous lines of evidence, together with our observed strong association between sibling obesity and childhood obesity, collectively suggest that behavioral imitation among siblings, shared activity patterns, and the common family dietary environment play critical roles in the familial aggregation of obesity. Their influence may even exceed the behavioral modeling provided by parents. We acknowledge that, although we observed significant associations between family members' obesity and childhood obesity (Figures 2A, B), our cross-sectional study design did not allow us to directly measure genetic predispositions or objectively assess family behavioral interactions. Therefore, the genetic and behavioral pathways discussed above remain speculative. The observed associations may reflect shared genetic, environmental, or behavioral factors, rather than definitively indicating specific causal mechanisms.

Grandparental obesity status showed no significant association with childhood obesity (Figures 2A, B), indicating that while three generation households are common, the nuclear family, consisting of parents and children, remains the primary environmental unit shaping childhood weight. This implies that lifestyle modifications targeting the nuclear family unit may be a more efficient strategy than cross generational interventions. The reverse gradient of parental education further supports the dramatic role of socioeconomic disparities. In the present study, as childhood obesity severity increased, the proportion of parents with primary school education or below markedly rose, while the proportion of those with junior high school education or above correspondingly declined (Figures 2C, D). This finding is consistent with multiple international studies. A study in German preschool children showed that children from families without a higher education background had twice the probability of overweight compared with those from higher education families, and the prevalence of overweight was 11.9% and 3.7%, respectively, a significant difference (27). A systematic review and meta-analysis across low, middle, and high income countries further revealed a more complex picture. In low and middle income countries, fruit and vegetable intake showed a stronger protective effect against childhood overweight and obesity, whereas low parental education significantly increased obesity risk only in high income countries (28). The gradient trend of parental education and obesity severity observed in our study is consistent with these findings, suggesting that in countries undergoing nutritional transition such as China, low education level may increase obesity risk through mediating pathways including unhealthy feeding practices, lack of nutritional knowledge, high stress, and irregular daily routines. This finding emphasizes that public health nutrition recommendations should clearly distinguish between education and resource inequalities that need to be addressed at the policy level and opportunities for individual behavioral change.

This study simultaneously described graded changes across multiple meal occasions, including breakfast, lunch, dinner, and snacks, within the same sample. A prominent pattern was a continuous decline in breakfast frequency with increasing obesity severity (Figures 3A, B). The findings of a cross-sectional study conducted among university teachers in Peru are consistent with the results of the present study focusing on children. Individuals with lower breakfast frequency or breakfast skipping exhibited an elevated risk of overweight and obesity, as well as poorer cardiometabolic indicators including waist circumference, blood pressure, blood glucose, and blood lipids (29). These observations suggest that low breakfast frequency is closely associated with a higher cardiometabolic risk profile, and that regular breakfast consumption may serve as an important lifestyle factor to reduce cardiometabolic risk and obesity. Further, regarding dietary diversity, Xu et al. (30) using longitudinal data from China demonstrated that low dietary diversity based on recommended food groups was significantly associated with an increased risk of overweight or obesity in Chinese children, and that low dietary diversity predicted higher body weight, higher body mass index, and a higher percentage of body fat. The present study further extends this finding to the level of specific meals. The median number of food types consumed at lunch and dinner was significantly reduced in children with severe obesity, suggesting that a monotonous dietary pattern restricts the intake of essential micronutrients and dietary fiber. We acknowledge that differences in vegetable, fruit, legume, seafood, and poultry consumption across obesity severity groups may partly reflect variations in total energy intake rather than the protective effects of these food groups *per se*, as we did not assess total caloric consumption. Our food frequency questionnaire was designed to capture consumption frequency of food groups rather than quantitative portion sizes or total energy intake, and consequently we were unable to adjust for overall energy intake in the multivariable models. Similarly, we acknowledge that socioeconomic status is a well established confounder in childhood obesity research, and while we adjusted for parental education in our models, this may not fully capture the broader socioeconomic context, as our questionnaire did not include detailed SES indicators beyond parental education level.

Regarding sleep, delayed bedtime was identified as a significant independent risk factor (Figures 8A, B). The meta analysis of observational studies by Hu et al. further consolidated this finding, demonstrating that late bedtime was significantly associated with obesity in children and adolescents, and this association remained robust after adjusting for sleep duration (31). Sensitivity analyses indicated that this association was not influenced by study geographic region, study design, participant age, or obesity diagnostic method. Additional reports have consistently shown that insufficient sleep, late sleep onset, poor sleep quality, and disrupted sleep rhythms are significantly and consistently associated with an increased risk of childhood obesity, and these associations are independent of other confounding factors such as diet and physical activity (32).

Several limitations of this study should be acknowledged in addition to those already discussed above. First, the cross-sectional design precludes any causal inferences or temporal conclusions regarding the observed associations. Second, dietary, sleep, and physical activity information was collected via parental or caregiver report, which may have introduced recall bias. Third, although we adjusted for multiple covariates, important potential confounders, including screen time, household income, psychological factors, and genetic predisposition, were not fully measured or accounted for. Residual confounding may therefore persist. Fourth, the study sample was drawn exclusively from Fujian Province. While it is representative of urban Han Chinese children in southern China, caution should be exercised when generalizing our findings to other regions or populations across the country. Fifth, certain behavioral variables, such as physical activity intensity, sleep quality, and quantitative dietary intake, lacked objective measurement tools, which may have affected the accuracy of exposure assessment. Regarding multiple comparisons, for the ordinal logistic regression models examining the associations of various exposure domains with childhood obesity severity, we did not perform additional correction for multiple testing (Figures 2B, 4H, 5H, 6C, 7B, 8C). These regression analyses were primarily exploratory and hypothesis-generating in nature, rather than confirmatory. Given the large number of variables tested, we acknowledge that some significant findings may have arisen by chance. Therefore, rather than relying solely on individual *P*-values, we have placed greater emphasis on consistent patterns and directional trends across related variables. Future studies incorporating longitudinal designs, objective measurements, and more comprehensive socioeconomic and psychological evaluations are warranted to further validate our findings.

## Data Availability

The original contributions presented in the study are included in the article/supplementary material, further inquiries can be directed to the corresponding author.

## References

[1] O'Connor SG Maher JP Belcher BR Leventhal AM Margolin G Shonkoff ET . Associations of maternal stress with children's weight-related behaviours: a systematic literature review. Obes Rev. (2017) 18:514–25. doi: 10.1111/obr.12522 28296057 PMC5523809

[2] Baker JL Olsen LW Sorensen TI. Childhood body-mass index and the risk of coronary heart disease in adulthood. N Engl J Med. (2007) 357:2329–37. doi: 10.1056/NEJMoa072515 18057335 PMC3062903

[3] Arisaka O Ichikawa G Koyama S Sairenchi T. Childhood obesity: rapid weight gain in early childhood and subsequent cardiometabolic risk. Clin Pediatr Endocrinol. (2020) 29:135–42. doi: 10.1297/cpe.29.135 33088012 PMC7534524

[4] Andersen LG Angquist L Eriksson JG Forsen T Gamborg M Osmond C . Birth weight, childhood body mass index and risk of coronary heart disease in adults: combined historical cohort studies. PLoS ONE. (2010) 5:e14126. doi: 10.1371/journal.pone.0014126 PMC2993956 21124730

[5] Bell KA Wagner CL Feldman HA Shypailo RJ Belfort MB. Associations of infant feeding with trajectories of body composition and growth. Am J Clin Nutr. (2017) 106:491–8. doi: 10.3945/ajcn.116.151126 28659299 PMC5525119

[6] Qiao J Dai LJ Zhang Q Ouyang YQ. A meta-analysis of the association between breastfeeding and early childhood obesity. J Pediatr Nurs. (2020) 53:57–66. doi: 10.1016/j.pedn.2020.04.024 32464422

[7] Rzehak P Oddy WH Mearin ML Grote V Mori TA Szajewska H . Infant feeding and growth trajectory patterns in childhood and body composition in young adulthood. Am J Clin Nutr. (2017) 106:568–80. doi: 10.3945/ajcn.116.140962 28659295

[8] Tian YS Ma XY Yang XZ Yin HZ Wang Y Long CJ . Prevalences and risk factors of overweight and obesity among kindergarten children in Shenzhen, China: a cross-sectional study. Front Public Health. (2026) 14:1780223. doi: 10.3389/fpubh.2026.1780223 PMC13013290 41889627

[9] Gupta S Lal P. Sleep and obesity risk in children: a systematic review of multiple dimensions of sleep. Children. (2025) 12:1577. doi: 10.3390/children12121577 41462718 PMC12731837

[10] Maia C Braz D Fernandes HM Sarmento H Machado-Rodrigues AM. The impact of parental behaviors on children's lifestyle, dietary habits, screen time, sleep patterns, mental health, and BMI: a scoping review. Children. (2025) 12:203 doi: 10.3390/children12020203 40003305 PMC11854690

[11] Mahmood L Flores-Barrantes P Moreno LA Manios Y Gonzalez-Gil EM. The influence of parental dietary behaviors and practices on children's eating habits. Nutrients. (2021) 13:1138. doi: 10.3390/nu13041138 33808337 PMC8067332

[12] Anzman SL Rollins BY Birch LL. Parental influence on children's early eating environments and obesity risk: implications for prevention. Int J Obes. (2010) 34:1116–24. doi: 10.1038/ijo.2010.43 20195285

[13] Tomayko EJ Tovar A Fitzgerald N Howe CL Hingle MD Murphy MP . Parent involvement in diet or physical activity interventions to treat or prevent childhood obesity: an umbrella review. Nutrients. (2021) 13:3227. doi: 10.3390/nu13093227 PMC8464903 34579099

[14] Kuswara K Shrewsbury VA Macdonald JA Chung A Hill B. Sharing the motherload: a review and development of the CO–parent conceptual model for early childhood obesity prevention. Obes Rev. (2024) 26:e13853. doi: 10.1111/obr.13853 39419655 PMC11711075

[15] Rae DE Tomaz SA Jones RA Hinkley T Twine R Kahn K . Sleep and BMI in South African urban and rural, high and low-income preschool children. BMC Public Health. (2021) 21:571. doi: 10.1186/s12889-021-10591-5 33757479 PMC7986550

[16] Slykerman RF Budd C Thompson JMD Bekker M Buckley J Wall C . Physical activity, sleep, body mass index, and associated risk of behavioral and emotional problems in childhood. J Dev Behav Pediatr. (2020) 41:187–94. doi: 10.1097/DBP.0000000000000754 32004246

[17] Nishioka T Hasunuma H Okuda M Taniguchi N Fujino T Shimomura H . Effects of screen viewing time on sleep duration and bedtime in children aged 1 and 3 years: Japan environment and children's study. Int J Environ Res Public Health. (2022) 19:3914. doi: 10.3390/ijerph19073914 35409594 PMC8997461

[18] Lister NB Baur LA Felix JF Hill AJ Marcus C Reinehr T . Child and adolescent obesity. Nat Rev Dis Primers. (2023) 9:24. doi: 10.1038/s41572-023-00435-4 37202378

[19] Tuerxun P Xu K Wang M Wei M Wang Y Jiang Y . Obesogenic sleep patterns among Chinese preschool children: a latent profile and transition analysisof the association sleep patterns and obesity risk. Sleep Med. (2023) 110:123–31. doi: 10.1016/j.sleep.2023.07.031 37574612

[20] Martínez-Gómez J Fernández-Alvira JM de Cos-Gandoy A Bodega P de Miguel M Tresserra-Rimbau A . Sleep duration and its association with adiposity markers in adolescence: a cross-sectional and longitudinal study. Eur J Prev Cardiol. (2023) 30:1236–44. doi: 10.1093/eurjpc/zwad137 37140006

[21] Danial B Faresjö T Fredriksson M Ludvigsson J. Childhood sleep and obesity risk: a prospective cohort study of 10000 Swedish children. Pediatr Obes. (2023) 18:e12983. doi: 10.1111/ijpo.12983 36222079 PMC10909437

[22] Jebeile H Kelly AS O'Malley G Baur LA. Obesity in children and adolescents: epidemiology, causes, assessment, and management. Lancet Diabetes Endocrinol. (2022) 10:351–65. doi: 10.1016/S2213-8587(22)00047-X 35248172 PMC9831747

[23] Chaput JP Gray CE Poitras VJ Carson V Gruber R Olds T . Systematic review of the relationships between sleep duration and health indicators in schoolaged children and youth. Appl Physiol Nutr Metab. (2016) 41(6 Suppl 3):S266–82. doi: 10.1139/apnm-2015-0627 27306433

[24] Chen H Wang LJ Xin F Liang G Chen Y. Associations between sleep duration, sleep quality, and weight status in Chinese children and adolescents. BMC Public Health. (2022) 22:1136. doi: 10.1186/s12889-022-13534-w 35668374 PMC9172025

[25] Liu M Cao B Luo Q Wang Q Liu M Liang X . Associations between sleep duration, wake-up time, bedtime, and abdominal obesity: results from 9559 Chinese children aged 7–18 years. Front Endocrinol. (2021) 12:735952. doi: 10.3389/fendo.2021.735952 34721295 PMC8552807

[26] Firman N Wilk M Marszalek M Griffiths L Harper G Dezateux C. Is obesity more likely among children sharing a household with an older child with obesity? Cross-sectional study of linked National Child Measurement Programme data and electronic health records. BMJ Paediatr Open. (2024) 8:e002533. doi: 10.1136/bmjpo-2024-002533 38599801 PMC11015308

[27] Hermeling L Steinacker JM Kobel S. Beyond correlates: the social gradient in childhood overweight. Arch Public Health. (2024) 82:3. doi: 10.1186/s13690-023-01232-x 38195594 PMC10775653

[28] Liu XT Wang YD Xu YJ Wang XY Shan SF Xiong JY . The divergent association of diet intake, parental education, and nutrition policy with childhood overweight and obesity from low- to high-income countries: a meta-analysis. J Glob Health. (2024) 14:04215. doi: 10.7189/jogh.14.04215 39582245 PMC11586647

[29] Saintila J Baquedano-Santana LE Calizaya-Milla YE Ramos-Vera C Barrientos AS Carranza-Esteban RF. Association between frequency of breakfast consumption and cardiometabolic risk in Peruvian university teachers, 2019-2020. Front Nutr. (2023) 10:1238223. doi: 10.3389/fnut.2023.1238223 37575324 PMC10419266

[30] Xu H Du S Liu A Zhang Q Ma G. Low dietary diversity for recommended food groups increases the risk of obesity among children: evidence from a Chinese longitudinal study. Nutrients. (2022) 14:4068. doi: 10.3390/nu14194068 36235720 PMC9571432

[31] Hu N Wu Y Yao Q Huang S Li W Yao Z . Association between late bedtime and obesity in children and adolescents: a meta-analysis. Front Pediatr. (2024) 12:1342514. doi: 10.3389/fped.2024.1342514 38560399 PMC10978672

[32] Gupta S Lal P Sharma R Gupta A Chaudhary BR. Systematic review of parental influence on pediatric obesity: exploring dietary habits, physical activity, and intervention strategies. Obes Pillars. (2025) 16:100218. doi: 10.1016/j.obpill.2025.100218 41281905 PMC12637252

[33] Formisano A Hunsberger M Bammann K Vanaelst B Molnar D Moreno LA . Family structure and childhood obesity: results of the IDEFICS Project. Public Health Nutr. (2014) 17:2307–15. doi: 10.1017/S1368980013002474 24053908 PMC10282634

